# Understanding the implementation of complex interventions in health care: the normalization process model

**DOI:** 10.1186/1472-6963-7-148

**Published:** 2007-09-19

**Authors:** Carl May, Tracy Finch, Frances Mair, Luciana Ballini, Christopher Dowrick, Martin Eccles, Linda Gask, Anne MacFarlane, Elizabeth Murray, Tim Rapley, Anne Rogers, Shaun Treweek, Paul Wallace, George Anderson, Jo Burns, Ben Heaven

**Affiliations:** 1 Institute of Health and Society, Newcastle University, 21 Claremont Place, Newcastle upon Tyne, NE2 4AA, UK; 2 General Practice and Primary Care, University of Glasgow, Glasgow, UK; 3 Agenzia Sanitaria Regionale – Regione Emilia Romagna, Bologna, Italy; 4 School of Population, Community and Behavioural Sciences, University of Liverpool, Liverpool UK; 5 National Primary Care Research and Development Centre, University of Manchester, Manchester, UK; 6 Department of General Practice, Clinical Science Institute, National University of Ireland, Galway, Ireland; 7 Department of Primary Care and Population Sciences, Royal Free and University College Medical School, London, UK; 8 Community Health Sciences Division, University of Dundee, Dundee, UK; 9 Nasjonalt Kunnskapssenter for Helsetjenesten, Oslo, Norway; 10 UK Clinical Research Network, London, UK

## Abstract

**Background:**

The Normalization Process Model is a theoretical model that assists in explaining the *processes *by which complex interventions become routinely embedded in health care practice. It offers a framework for process evaluation and also for comparative studies of complex interventions. It focuses on the factors that promote or inhibit the routine embedding of complex interventions in health care practice.

**Methods:**

A formal theory structure is used to define the model, and its internal causal relations and mechanisms. The model is broken down to show that it is consistent and adequate in generating accurate description, systematic explanation, and the production of rational knowledge claims about the workability and integration of complex interventions.

**Results:**

The model explains the normalization of complex interventions by reference to four factors demonstrated to promote or inhibit the operationalization and embedding of complex interventions (interactional workability, relational integration, skill-set workability, and contextual integration).

**Conclusion:**

The model is consistent and adequate. Repeated calls for theoretically sound process evaluations in randomized controlled trials of complex interventions, and policy-makers who call for a proper understanding of implementation processes, emphasize the value of conceptual tools like the Normalization Process Model.

## Background

Complex interventions – consisting of multiple behavioural, technological, and organizational components – are common and important features of health care practice and research. However, they pose special evaluation problems because their components may act independently or interdependently, and it is often difficult to tease out the relationships between them [1]. This has led to well documented difficulties in evaluating such interventions [1-4]. Process evaluations have therefore become an important focus of interest amongst trials designers and health services researchers. While trials and other outcomes studies focus on the clinical and cost effectiveness of complex interventions, process evaluations help to understand how those outcomes are reached, and the factors that promote or inhibit them.

In this paper, we focus on understanding the processes involved in implementing complex interventions. This is more than the adoption and diffusion of innovations [5,6]. Effective implementation means that complex interventions are made workable and integrated in everyday health care practice. This paper adds to the literature on understanding the processes of implementation by further developing a theoretical framework for understanding and evaluating them. This theoretical framework – the Normalization Process Model [7,8] – provides a tool that assists process evaluation in two ways. First, the model identifies and describes factors that have been shown to be important in promoting or inhibiting the implementation of complex interventions. Second, the model provides a basis for assessing the probability of a complex intervention to become routinely incorporated in practice.

Earlier papers described the key features of the model, and the methods by which it was derived from empirical studies [7], and illustrated its application by examining processes within two trials of complex interventions [8]. In this paper we advance the model by doing two things. First, we develop a simplified version of the model and show that it is a robust and effective sensitizing scheme for research questions. Second, we show that the model is consistent and adequate as an applied theory, and thus can be used to frame hypotheses about the outcomes of normalization processes. Both of these tasks are important if the model is to be used to inform empirical investigations. In particular, the paper makes clear what the model can, and cannot, be expected to achieve when employed as an analytic tool in empirical research. This is necessary to inform further tests and elaboration of the model.

## Methods

To be useful, a theoretical model must be both adequately described and fit for purpose. The Normalization Process Model provides a theoretical framework for understanding complex interventions. By this we mean that it provides transparent and transferable explanations for phenomena revealed by empirical investigation [9,10]. Such a framework allows investigators and others to evaluate the likely generalizability of explanations to other situations or contexts, and explain the congruence, or not, between predicted and observed phenomena.

Let us start by outlining the criteria for an adequate description of a theory. In this paper, we follow the formal tradition of theory building in sociology [11-13], and define a theory as a body of related ideas that forms the foundation for three kinds of conceptual work: describing, explaining and predicting observed phenomena.

*1. Accurate description*. A theory must provide a taxonomy or set of definitions that enable the identification, differentiation, and codification of the qualities and properties of cases and classes of phenomena.

*2. Systematic explanation*. A theory must provide an explanation of the form and significance of the causal and relational mechanisms at work in cases or classes of the phenomena defined by the theory, and should propose their relation to other phenomena.

*3. Knowledge claims*. A theory must lead to knowledge claims. These may take the form of abstract explanations, analytic propositions, or experimental hypotheses. They may also map relations with other phenomena that are believed to possess similar qualities and properties.

A fourth, but not mandatory, component of a theory is that it proposes a means of testing its knowledge claims:

*4. Investigation*. A theory must be testable. Such tests may be abstract (i.e. formal logical representations, simulations, or thought experiments); or concrete (empirical investigations).

To be fit for purpose a theory must therefore do more than describe a set of phenomena. It must also explain their operation. Such explanations may take three forms [13]. (i) Causal explanations deal with phenomena where one thing acts upon another: e.g. where the force of gravity acts upon an apple to make it fall to the ground. (ii) Transformative explanations deal with phenomena where one thing interacts with another: e.g. where mixing two chemical compounds causes a reaction that makes a third. (iii) Relational explanations deal with phenomena where the presence or absence of one thing leads to a change in another: e.g. where a person with visual impairment experiences more acute hearing. In this context, the task of the model is to describe and explain the work by which complex interventions are enacted and embedded by individuals and groups working in healthcare and related settings.

## Results and Discussion

The Normalization Process Model proposes that evaluating the implementation of complex interventions requires attention to more the measurement of outcomes and effectiveness, but also to the social relations and processes related to the *work *that leads to those outcomes. In particular, it guides attention to the processes by which complex interventions are made *workable *and *integrated *in everyday practice. Above, we defined the requirements of a theory. In the following section, we will describe the components of the Normalization Process Model, and show how it meets those requirements.

### Description: what are the phenomena to be explained?

The model focuses on phenomena that are the products of co-operative and collective activities, but which are experienced and accounted for by individuals. Theories of individual *preferences *(in economics [14]), *intentions *(in psychology [15]), and *interests *(in sociology [16]) help us to understand how participants in these collective activities frame behaviour. Because such theories focus on individual and not group processes, they are inevitably much less successful in accounting for *organizational *processes characterized by complexity and emergence, where multiple confounders act upon behaviour. The Normalization Process Model is concerned with explaining those factors that promote or inhibit the implementation of complex interventions by reference to collective social action, and draws extensively on sociological research on group processes in structured organizational contexts. It thus includes those multiple confounders in its frame of reference. The unit of analysis of the model is therefore group processes leading to collective action.

In this context, a complex intervention is defined as a deliberately initiated attempt to introduce new, or modify existing, patterns of collective action in health care. Deliberate initiation means that an intervention is: institutionally sanctioned; formally or informally defined; consciously planned; and intended to lead to a changed outcome. Initiators of a complex intervention may seek to change the ways that people think, act and organize themselves in health care, or they may seek to initiate a process with the intention of creating a new outcome. There are three core components of such interventions:

(i) *Actors *are the individuals and groups that encounter each other in health care settings. Examples are health professionals, hospital managers and patients. Complex interventions aimed at individuals and groups may take the form of attempts to change the ways that people behave, for example, in trials of strategies for making 'expert patients' [17]; or they may take the form of a new ways of defining, classifying, and speaking about a problem, for example, in therapeutic attempts to recast the experience of chronic pain [18]. The aims of such interventions are often to change people's *behaviour *and its intended *outcomes*.

(ii) *Objects *are the institutionally sanctioned means by which knowledge and practice are enacted. Examples are established drug therapies, trial protocols, clinical guidelines and electronic medical records. Complex interventions relating to objects include trials of novel therapeutic agents and medical devices [19], and of decision-making tools and clinical guidelines [20]. The aims of such interventions often include changing people's *expertise *and *actions*.

(iii) *Contexts *are the physical, organisational, institutional, and legislative structures that enable and constrain, and resource and realize, people and procedures. Complex interventions relating to context include trials of new professional roles, mechanisms that mediate between health care organisations and professional groups, and organisational structures. The aims of such interventions are often to change the ways that people enact *procedures *to achieve *goals *in health care (or other) settings.

In relation to these components we must distinguish between normalization as an *accomplishment*, and normalization as a possible *outcome *of that accomplishment. A normalization process consists of the collective action – the *work *– involved in enacting a complex intervention. When that work leads to the routine embedding of an intervention in everyday practice, it may be said to have become normalized. Normalization does not, however, imply an evaluation of effectiveness or quality.

Normalization is only one possible outcome of collective action. Others include: *adoption*, where a complex intervention is taken up but does not become routinely embedded in everyday work; and *rejection*, where users disregard, subvert, or otherwise refuse a complex intervention. Thus normalization is not automatically the outcome of the initiation of a new or changed set of practices. De-normalization may also occur during the lifetime of a complex intervention when a previously normalized intervention is superseded, disturbed, disrupted, or atrophied. Thus normalization is neither an automatic outcome nor a permanent state.

### Explanation: how does normalization come about?

The model is constrained by its focus on *work *as collective action, over time, in health care settings. It is based on three assumptions. First, the model assumes implementation. This is defined as a pattern of organized, dynamic, and contingent interactions in which individuals and groups work with a complex intervention, within a specific context or health system, over time. Second, the model assumes a set of factors empirically demonstrated to affect the outcome of the process. These four factors – defined below as constructs of the model – each have two dimensions: (a) *Co-operative *attributes that are oriented towards enacting the intervention through negotiations and agreements between people and the organizations and policymakers providing the context within which they work; and (b) *Executive *attributes that are oriented to attempts to project enacting the intervention outwards in time and space. The constructs and their dimensions are:

1. *Interactional Workability *– how does a complex intervention affect interactions between people and practices?

*a. Congruence *is concerned with interaction itself: what can legitimately be dealt with in an interaction (e.g. a consultation), what the form of the work is, what the role of each participant is, how the work is to be completed in the time and space available, and the formal and informal rules that govern the verbal and non-verbal conduct of an interaction.

*b. Disposal *of work is concerned with the effects of interactions. It considers the goals of an interaction (e.g. following a guideline, recording or processing data), how disagreement about the outcome of the work is minimised, when and where the goals and outcomes should occur, and shared beliefs about the meaning and consequences of the work. It can also relate to the interaction between the human and non-human actors (e.g. using a computer programme). For all these interactions whether the intervention promotes the ease/efficiency of the interaction is a key feature.

2. *Relational Integration *– how does a complex intervention relate to existing knowledge and relationships?

*a. Accountability *is concerned with the knowledge and practices of those enacting the complex intervention, what is the knowledge required by the work, who has this knowledge, are there disagreements about where (and with whom) the necessary knowledge lies, what contributions are required of participants, and what are the formal and informal rules that govern the distribution of knowledge and practice within relational networks.

*b. Confidence *refers to beliefs about the knowledge and practice required by a complex intervention. It considers agreement about the sources of authoritative knowledge and practice, the criteria by which their credibility can be assessed, and beliefs about the practical utility and reliability of the knowledge and practice mediated by the various networks in the health system. So for example, the perceived safety of the intervention is important.

3. *Skill-set Workability *– how is the current division of labour affected by a complex intervention?

*a. Allocation *is concerned with which tasks are performed by whom and how these decisions are made (e.g. whether a healthcare innovation is more appropriately used by a doctor or a nurse), the distribution of resources and rewards linked to status and authority, formal or informal agreements about the identification and appraisal of the necessary skills, and the definition and ownership of these skill-sets.

*b. Performance *considers the ability of an organisation and the people within it to effectively organise and deploy a complex intervention as part of their activities (e.g. do surgeons need extensive training to use a new piece of equipment?). It covers staff training needs, formal and informal policies that define the boundaries of competence of particular workers, the degree of autonomy these assigned to them, and how they deliver services.

4. *Contextual Integration *– how does a complex intervention relate to the organisation in which it is set?

*a. Execution *is concerned with the practicalities of integration (e.g. does the intervention require new money, a local or national policy sponsor), decisions about the distribution of resources, costs and risks within the organisation, managerial decision-making regarding the adoption of the intervention, and formal and informal mechanisms for its evaluation.

*b. Realisation *considers the allocation and ownership of responsibility for the implementation of a complex intervention (e.g. does the complex intervention require responsibility for a procedure to move from one professional group to another?), the negotiations necessary to modify existing systems and practices to make new ones possible, minimising the disruption and risk associated with change, and how new resources are obtained and used in practice.

Finally, it is assumed that variations in the outcome of an implementation process can be correlated to variations in the factors that affect its course. It is thus possible to determine the degree to which a complex intervention is ultimately normalized or not normalized, and to determine the probable degree to which specific factors affect outcomes.

### Knowledge claims and empirical investigations

Determining the effect of the factors identified by the model may be undertaken by means of objective measures, or subjective investigations. Four propositions derived from the model were presented an elaborated in earlier papers [7,8]. These propositions can be used as the basis of instruments to assess the effect of those factors that promote or processes and as the basis of hypotheses about the normalization *potential *of a complex intervention.

The practical utility of the model lies in the ability to make testable claims about the factors that promote or inhibit a complex intervention's potential for workability and integration in practice. The model is open to knowledge claims founded on empirical investigation. These may take its core constructs and test them retrospectively to make claims about processes with already known outcomes, or prospectively test them against processes where the outcome is yet to be determined. Here, even though variations in outcome may be correlated with observed variations in the factors defined by the model's constructs, the *mechanisms *by which those factors affect normalization outcomes must be described if the model is to be useful. These mechanisms are defined by reference to the dimensions that obtain to each of the core constructs of the model outlined above

The practical utility of the model depends on its adequate explanation of a set of complex, and contingent, social relations and processes at work in health care settings. Because this is a sociological approach that attends to the construction and embedding of practices by focusing on *what the work is*, how it is known, allocated and resourced – the starting point for empirical investigation is collective action. Such investigations will therefore involve modelling and mapping the relations between people, objects and contexts; understanding their conditions of action (defined as processes); and observing or measuring the effects of the factors that govern these (defined above as constructs and dimensions of the model).

The goal of many implementation theories is the prediction of outcomes [21], and this is a significant methodological and theoretical challenge [9,22]. The Normalization Process Model generates hypotheses about the factors that affect the course of normalization processes. In real-world settings, predictions about outcomes are subject to multiple confounders that include complexity and emergence that lead to local variations in implementation processes. These confounders may include events or processes far beyond the purview of participants in the implementation of a complex intervention. Importantly, they include many external factors that are not amenable to control or modification. This means that predicting the course and outcome of complex social processes is problematic [13]. The Normalization Process Model is not excepted from this rule. However, although absolute prediction is outside of the field of application for the model, the *probability *of a practice to normalize can be calculated within limits. This means that claims about the future of a complex intervention must take the form of assessments of the potential of a practice to normalize in a specific setting, and of the readiness of actors to accept it.

## Conclusion

Applied theories are not intended to be ornamental. They are tools to be used to make descriptions, explanations and investigations within certain limits. The Normalization Process Model is therefore aimed at a limited range of phenomena – specifically, the implementation of complex interventions in healthcare settings in relation to the *work *that it involves. The limits of the model mean that it is not intended to deal with two problems:

(i) *Diffusion and adoption: *The diffusion of innovations across networks of organizations or organizational units, and their adoption by individual or collective 'champions' [5,21] is the proper domain of diffusion of innovations theory.

(ii) *Intention and volition: *The mental components of individual behaviour, especially the cognitions and intentions that might dispose individuals to adopt a complex intervention [23,24], are the proper domain of psychological theory.

Although it is defined in strict terms in this paper, the model is *not *a general theory because it does not promise a set of universal laws about the routine embedding of practices in everyday life, but rather it works as an applied theoretical model that seeks to explain the embedding of complex interventions in healthcare settings. These are conceived as processes of purposive collective action (for example, by users of new technologies).

However, employing a strict definition of theory that gives a structure to the formal description of the model has meant that our account has taken a particular form. Principally, we have had to pretend for a moment that the dynamic components of a system are static and linear. However, the situations in which complex interventions are initiated are, of course, contingent, variable, dynamic and unevenly distributed. They are characterized by complexity and emergence. The emergent qualities of implementation processes mean that empirical rather than theoretical investigation is a vital part of the development of the model. Coherence, consistency and explanatory power therefore need to be maintained by establishing the proper range and scope of the theory and staying within it. In this context, simple instruments for calculating normalization scores could have great practical value, if the analytic constructs of the model – and the propositions derived from them – are underpinned by an adequate account of causal mechanisms at work.

Finally, in this paper we have set out the structure of the model as a practical tool. It provides an applied theory of normalization processes that defines and explains the routine embedding of complex interventions by reference to the work that people do in implementing and operationalizing a complex intervention. Further, it suggests the form of claims that can be made about this process, and directions for their investigation. In this paper, we have set the model out according to a formal definition of theory: but does it matter whether it meets these demands or not? If it is to be used only as a heuristic device then strict definitions of this kind are probably irrelevant to its users. But the key claim we have made about the Normalization Process Model is rather more than this. It is that it is a means of accomplishing three related tasks:

(i) *Descriptions: *The model systematically establishes and differentiates the phenomena with which it is concerned by defining actors, objects and contexts, and the processes that govern them. It therefore permits a rational foundation for explanations of observed events and processes pertaining to the implementation of complex interventions in health care systems.

(ii) *Explanations: *The model offers a systematic explanation of the operation of those processes and conditions by referring to patterns of collective action that can be empirically shown to affect their outcomes, and by defining the causal mechanisms and relations that underpin these. The model may thus be reasonably employed to make predictions about the normalization potential of proposed interventions, and about the possible outcomes of other implementation processes.

(iii) *Knowledge claims: *The model permits verifiable knowledge claims about process and action, and proposes a set of analytic propositions that can inform empirical investigation. This means that it not only accounts for outcomes of implementation processes, but can also account for differences between expected and observed outcomes of complex interventions in real health care settings.

The model explains routine embedding by reference to social processes. These processes are located in specific contexts and take place over time (that is, those who initiate them have an end in sight). The knowledge claims that are permitted by the model are not restricted to retrospective interpretative analysis or simulations, but may be developed and refined prospectively through investigation by experiment or observation. Repeated calls for theoretically sound process evaluations in randomized controlled trials of complex interventions [2,4,25], and policy-makers who call for a proper understanding of implementation processes [26], give emphasis to the value of conceptual tools like the Normalization Process Model.

## Competing interests

The author(s) declare that they have no competing interests.

## Authors' contributions

Authorship of this paper is attributed on the following basis. CRM led on the program of theory building represented in the paper, and with TF and FM, defined the core theoretical components of this paper. He also drafted the paper. LB, CD, ME, LG, AMacF, EM, TR, AR, ST and PW contributed to the theoretical and methodological elaboration of normalization processes. For resubmission, CRM, LB, EM, FM, TR and ST made modifications to the manuscript, and GA, JB and BH contributed additional material that assisted in the simplification of the model's constructs.

## Pre-publication history

The pre-publication history for this paper can be accessed here:


